# Small RNA Profiling in *Mycobacterium* Provides Insights Into Stress Adaptability

**DOI:** 10.3389/fmicb.2021.752537

**Published:** 2021-11-04

**Authors:** Yingyu Chen, Wenjun Zhai, Kailun Zhang, Han Liu, Tingting Zhu, Li Su, Luiz Bermudez, Huanchun Chen, Aizhen Guo

**Affiliations:** ^1^State Key Laboratory of Agricultural Microbiology, College of Veterinary Medicine, Huazhong Agricultural University, Wuhan, China; ^2^National Animal Tuberculosis Para-Reference Laboratory (Wuhan) of Ministry of Agriculture and Rural Affairs, Huazhong Agricultural University, Wuhan, China; ^3^Department of Biomedical Sciences, College of Veterinary Medicine, Oregon State University, Corvallis, OR, United States

**Keywords:** *Mycobacterium bovis*, *Mycobacterium bovis* BCG, sRNA, stress, mycobacteria

## Abstract

*Mycobacteria* encounter a number of environmental changes during infection and respond using different mechanisms. Small RNA (sRNA) is a post-transcriptionally regulatory system for gene functions and has been investigated in many other bacteria. This study used *Mycobacterium tuberculosis* and *Mycobacterium bovis* Bacillus Calmette-Guérin (BCG) infection models and sequenced whole bacterial RNAs before and after host cell infection. A comparison of differentially expressed sRNAs using Gene Ontology (GO) and Kyoto Encyclopedia of Genes and Genomes (KEGG) and target prediction was carried out. Six pathogenically relevant stress conditions, growth rate, and morphology were used to screen and identify sRNAs. From these data, a subset of sRNAs was differentially expressed in multiple infection groups and stress conditions. Many were found associated with lipid metabolism. Among them, ncBCG427 was significantly downregulated when BCG entered into macrophages and was associated with increased biofilm formation. The reduction of virulence possibility depends on regulating lipid metabolism.

## Introduction

*Mycobacterium tuberculosis* (*M. tb*) is the leading cause of human tuberculosis (TB), one of the top 10 most important causes of death worldwide (WHO, 2020). *M. tb* complex, especially *M. tb* and *Mycobacterium bovis*, are also the leading cause of animal TB (Sun et al., 2019), and are responsible for major economic losses, therefore, representing a great threat to public health. Although zoonotic TB is an old disease, afflicting both humans and animals for thousands of years, it still remains a major health emergency.

Small RNAs (sRNAs) are post-transcriptional regulators which play very important roles in the translation and/or mRNA stability during bacterial infections (Gong and Klumpp, 2017; Jorgensen et al., 2020). The number of investigations about the roles of sRNA in infection has been increasing, although mycobacterial sRNA associated with macrophage infection, particularly *M. tb*, has not been much addressed.

The pathogenicity of *M. tb* can be affected by many variables, such as virulence factors of the bacterium (Bychenko et al., 2021), expression of host genes (Madhavan et al., 2021), and cross-talk between the pathogen and host cells (Sun et al., 2020). The *M. tb* cell wall contains many complex lipids presenting as major effector molecules that interact with the host, modulating its metabolism and stimulating the immune response (Gago et al., 2018). In *M. tb*, about 250 genes are involved in lipid metabolism. They not only regulate the replication and persistence of the bacterium inside the host cells, but also influence cellular signaling, membrane micro domain organization and dynamics, and membrane trafficking (Rameshwaram et al., 2018). Therefore, lipid metabolism is an important component in the life cycle of *Mycobacterium*.

The ability to adapt to diverse stresses in host environments is essential for a successful *Mycobacterium* infection. When entering cells, the pathogen will certainly encounter huge environmental challenges (Bansal et al., 2017). Because RNA and protein syntheses are slow in slow-growing mycobacteria than rapid-growing microbes, a mechanism must exist to facilitate adaptation to rapidly changing host conditions. Given the relative paucity of information on the identity and function of sRNAs during mycobacterial infection, RNA-sequencing (RNA-seq) was used to comprehensively identify sRNAs differentially expressed by both *M. tb* and *M. bovis* BCG in intracellular and extracellular environments, under six relevant stress conditions. Of interest, from a subset of differentially expressed sRNAs, many were related to lipid metabolism. Among those, the expression of ncBCG427 is reduced during exposure to iron starvation, lower pH stress, and membrane stress.

This study determined the ability of *M. tb* sRNA to enhance biofilm growth and reduces bacterial virulence and investigated whether virulence possibility depends on the regulation of lipid metabolism.

## Materials and Methods

### Strains and Growth Conditions

*Mycobacterium tuberculosis* strain 1458 (GenBank accession no. NZ_CP013475.1), originally obtained from a cow, was isolated and stocked in this laboratory. *M. bovis* BCG (Tokyo strain, ATCC 35737) was kindly provided by Junyan Liu from Wuhan University. *Mycobacterium smegmatis* MC^2^ 155 strain (NC_008596.1) was donated by Luiz Bermudez from Oregon State University.

All *M. tb*-related experiments in this research were carried out strictly in accordance with the biosafety-related operating procedures in the Animal Biosafety Level 3 Laboratory of the State Key Laboratory of Agricultural Microbiology in Huazhong Agricultural University. *M. tb* 1458 and *M. bovis* BCG were cultured to mid-log phase in 20 ml Middlebrook 7H9 medium supplemented with 10% oleic acid, albumin, dextrose, and catalase medium and 0.05% Tween-80 and incubated at 37°C for 2 weeks.

### Cell Culture

THP-1 cells were kindly provided by Chuanyou Li from the Beijing Tuberculosis and Thoracic Tumor Research Institute and were cultured in RPMI-1640 medium supplemented with 10% heat-inactivated fetal bovine serum (FBS). THP-1 cells were seeded in 24-well tissue culture plates and treated with phorbol-ester (PMA; 5 μM) for 24 h to induce maturation. Cells were seeded at 90% confluence. Cell viability during the infection was monitored.

A549 human type II alveolar epithelial cells were kindly provided by Luiz Bermudez from Oregon State University and were maintained in Dulbecco’s modified Eagle’s medium supplemented with 10% heat-inactivated FBS. A total of 10^5^ cells were added to each well of a 24-well tissue culture plate. Experiments were carried out in 90% confluent monolayers.

### Bacterial Infection

PMA-differentiated THP-1 cells were infected with *M. tb* 1458 or *M. bovis* BCG at a multiplicity of infection (MOI) of 10 for 12 h. Supernatants that contained bacteria were collected as control for the number of extracellular bacterial.

The remaining cells were gently washed twice with phosphate-buffered saline (PBS). Infected cells were incubated for 45 min in media containing 50 μg/ml gentamicin (Sigma-Aldrich) to kill extracellular bacteria. After washing, THP-1 cell cultures were maintained in RPMI-1640 medium. When needed according to the experimental protocol, cells were scrapped from the flask and collected 6 and 24 h PI, by centrifugation at 200 × *g* for 10 min at 4°C.

### RNA Extraction and Sequencing

Extracellular bacterial total RNA was extracted using Trizol reagent (Invitrogen, United States) according to the manufacturer’s instructions. For the intracellular bacterial samples, bacteria were separated from eukaryotic cells at several time points, washed twice with RPMI-1640 medium, and added with GTC lysis solution. After rapid lysing, the preparation was centrifuged at 5,000 × *g* for 20 min, followed by 10,000 × *g* for 20 s after GTC resuspension. Tween-80 was added for 2 min, followed by RNA-free water. The total RNA of the intracellular bacterial sample was extracted using FastRNA Blue Kit (MP Biomedicals, Shanghai, China), according to the manufacturer’s instructions. The total RNA concentration was measured by an ultraviolet spectrophotometer at 260/280 nm (Thermo Fisher Scientific, United States). cDNA was synthesized and sent for sequencing after the quality-control test.

### Data Analysis and Annotation

FASTX toolkit was used to get clean reads and HTSeq was used to count the reads. The clean reads were aligned to *M. tb* 1458 (NZ_CP013475) and *M. bovis* BCG (NC_012207) genomes by using the Bowtie2 (Langmead and Salzberg, 2012) with no more than 1 nt mismatch. Aligned reads with more than one genome location were not considered. Uniquely localized reads, with (1) 40–500 bp peaks, (2) median height of a single peak with no less than 20 nt, and (3) maximum height of a single peak of not less than 60 nt, were screened.

Fold changes no less than 1.5 or existing only either extracellularly or intracellularly were regarded as differentially expressed non-coding RNAs (ncRNAs) in extracellular or intracellular bacterial samples. RPKM was used to compare ncRNAs in different groups.

Rfam^1^ and BRSD^2^ databases were used to annotate all sRNAs. DAVID^3^ was used for Gene Ontology (GO) enrichment analysis. KOBAS version 3.0^4^ was used for Kyoto Encyclopedia of Genes and Genomes (KEGG) pathway enrichment analysis. IntaRNA^5^, CopraRNA^6^, and TargetRNA2^7^ tools were used to predict sRNA target genes. Candidate genes predicted by all three tools were selected for further verification.

### Stress Culture Model

Iron starvation, carbon hunger, acidification pressure, oxidation pressure, membrane pressure model, and granuloma-like models were used, as described previously (Gerrick et al., 2018; Chen et al., 2019). Briefly, the iron starvation medium was additionally treated with Chelex-100 (10 g in 1,000 ml culture medium) to remove residual iron. The carbon hunger medium was PBS, pH 4.5 was employed for acidification pressure. Tert-butyl hydroperoxide (1 mM) or 0.05% sodium dodecyl sulfate was added to the 7H9 medium for oxidative stress and membrane stress model, respectively. Granuloma-like model used 7H9 medium with 0.3 M dextrose (pH 6.0) and cultured with no oxygen.

Following incubation, bacteria were spun down at 3,800 × *g* for 10 min, then bacteria were washed in PBS and resuspended in a 1:1 (v/v) stress culture medium and incubated at 5% CO_2_ for 24 h. Bacteria were collected for further use.

### Real-Time Polymerase Chain Reaction Analysis

RNA was extracted and reverse-transcribed to cDNA using HiScript II Q RT SuperMix for qPCR (Vazyme, China) or All-in-One^TM^ miRNA First-Strand cDNA Synthesis Kit (Genecopoeia, United States). *sigA* gene or *MysA* was used as an internal reference. The primers for amplification of selected genes are listed in Supplementary Table 1. RT-PCR was performed in 10 μl reaction mixtures, including 5 μl of 2 × SYBR Green Master Mix (Vazyme), 10 μM of forward and reverse primers, 0.2 μl Dye 2, 1.5 μl cDNA template (1:10 diluted), and double-distilled water to a final concentration of 10 μl. Samples and standards were run in triplicate in an ABI 7500 thermocycler (Applied Biosystems, United States) and analyzed using model 7500 SDS software version 1.3.1. PCR was carried out at 94°C for 10 min and then 94°C for 15 s and 60°C for 1 min for a total of 40 cycles.

### Overexpression of Bacterial Constructs

The rrnB promoter (between −200 and −8) was amplified from *M. smegmatis* and used to replace the Hsp60 promoter in pMV261 by digesting the DNA with *Xba*I-*Hin*dIII to generate pMV261-PrrnB. A seamless cloning technique was used to combine the rrnB promoter and the Term Transcriptional terminator.

All primers used in this study are listed in Supplementary Table 2 using the following systems: 98°C for 5 min then 98°C for 30 s, annealing for 30 s, and 72°C for 2 min, for a total of 30 cycles at 72°C for 10 min. The PCR-generated fragments were cloned into a pMV261-PrrnB vector encoding kanamycin resistance. The resulting plasmids were propagated into DH5α *Escherichia coli* bacteria and electroporated into *M. smegmatis* MC^2^ 155 or BCG. Transformants were selected from Middlebrook 7H11 agar plates containing 50 μg/ml kanamycin and screened by PCR using the primers originally used for PCR. The obtained fragment was then sequenced.

### Growth Curve, Morphology

The recombinant strains were cultured in a 7H9 medium with 50 μg/ml kanamycin in 37°C and taken every 12 h for 7 days to draw the growth curve. The morphology of a single colony was observed and recorded under a stereomicroscope. The area of every single colony was calculated by the three-point rounding method. Five to ten single colonies were recorded in each plate.

### Biofilm-Forming Ability

The recombinant strains were cultured to log phase (OD_600__nm_ = 0.6–0.8), and bacteria were collected after centrifugation and resuspended and dispersed in fresh 7H9 medium, adjusted to OD_600__nm_ = 1. Bacteria were diluted at 1:100 in fresh Sauton medium with kanamycin, inoculated into a 12-well plate, sealed with a sterile membrane, and incubated at 37°C for 5–7 days. The biofilm formation thickness of each strain was recorded. Three repeat groups were set up for each strain.

The liquid and bacteria outside the biofilm were removed, the plate was completely dried, and 1% crystal violet dye was added for 10 min. The plate was completely dried after washing, 95% alcohol was added for 10 min after fully dissolved, OD_570__nm_ was read after three times dilution, and three repeat holes were set. The differences in biofilm formation thickness and absorbance were compared.

### Statistical Analysis

*In vitro* experiments were repeated at least thrice. Analysis of variance (ANOVA) was used to analyze more than two groups, whereas Student’s *t*-test was used to analyze two groups. *p* < 0.05 was considered significant. GraphPad Prism version 7.0 software was used for statistical analysis.

## Results

### Comparison of Non-coding RNA From Extracellular and Intracellular Bacterial Preparations

An analysis of differentially expressed ncRNAs in different bacterial preparations was performed. There were 490 ncRNAs in BCG compared to 349 ncRNAs in *M. tb* 1458. Moreover, 247 (50.4%) ncRNAs were expressed both intracellularly and extracellularly in BCG; 76 (15.5%) were expressed only intracellularly and 171 (34.9%) were expressed only extracellularly; 133 (38.1%) ncRNAs were expressed both intracellularly and extracellularly in *M. tb* 1458, 158 (45.3%) were expressed only intracellularly and 58 (16.6%) were expressed only extracellularly. Total reads number and proportion of extracellular and intracellular bacterial fractions that were mapped to the reference genome were listed in Supplementary Table 3.

**FIGURE 1:**
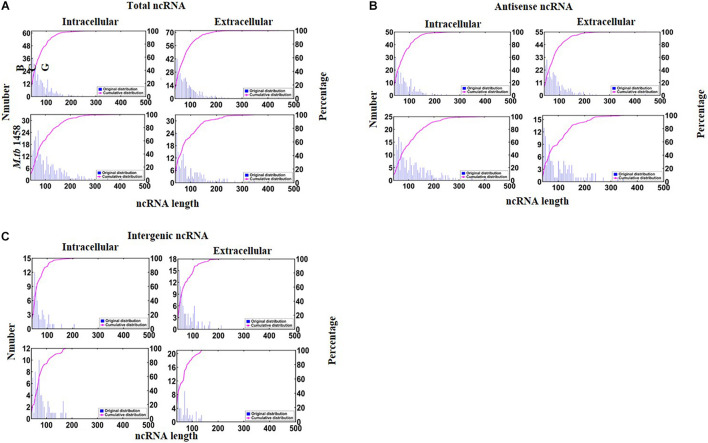
Distribution of ncRNA length: **(A)** total, **(B)** antisense, and **(C)** intergenic ncRNAs.

There were 190 antisense and 53 intergenic ncRNAs successfully mapped in both BCG groups compared to 91 antisense and 42 intergenic ncRNAs successfully mapped in two *M. tb* 1458 groups. Moreover, 323 ncRNAs, including 254 antisense and 69 intergenic ncRNAs, were uniquely expressed in intracellular BCG, and 417 ncRNAs, including 323 antisense and 94 intergenic ncRNAs, were uniquely expressed in extracellular BCG. In intracellular *M. tb* 1458, there were 297 ncRNAs, including 223 antisense and 74 intergenic ncRNAs. In extracellular *M. tb* 1458, there were 193 ncRNAs, including 126 antisense and 67 intergenic ncRNAs. After a comparison of the length distribution of the different ncRNA groups, for 0–200-nt-long ncRNA, the extracellular group had much more ncRNAs than the intracellular group, for BCG, but the extracellular group had much lower numbers in *M. tb* 1458 (Figure 1A). The antisense and intergenic ncRNA length was determined. ncRNAs less than 200 nt for both antisense and intergenic ncRNAs were more than intracellular ncRNAs in BCG. The *M. tb* 1458 group had opposite results and had lower intergenic ncRNA in extracellular bacteria. Compared to BCG ncRNA with less than 150 nt, *M. tb* 1458 ncRNAs were mainly less than 100 nt (Figures 1B,C).

### Differentially Expressed Non-coding RNAs in Different Bacterial Groups

**FIGURE 2:**
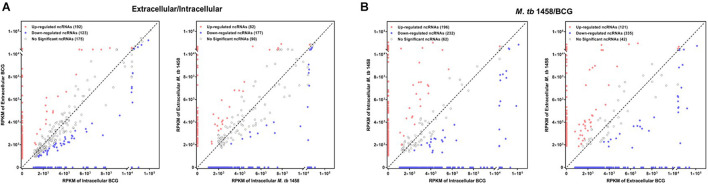
RPKM distribution of ncRNAs: **(A)** *Mycobacterium tuberculosis* 1458/BCG RPKM distribution of ncRNAs between intracellular and extracellular bacteria (| FC| > 1.5). **(B)** RPKM distribution of ncRNAs between BCG and *M. tb* 1458 (| FC| > 1.5).

Fold changes no less than 1.5 or existing only either extracellularly or intracellularly were regarded as differentially expressed ncRNAs in extracellular or intracellular bacterial samples. RPKM was used to compare ncRNAs in different groups. Compared to the intracellular bacterial sample, 192 ncRNAs were upregulated and 123 were downregulated in extracellular BCG and 82 ncRNAs were upregulated and 177 were downregulated in extracellular *M. tb* 1458 (Figure 2A). Fold changes no less than 1.5 or existing only in either BCG or *M. tb* 1458 were regarded as differentially expressed ncRNAs between BCG and *M. tb* 1458. RPKM was used to compare ncRNAs in different groups. Compared to intracellular BCG, 196 ncRNAs were upregulated and 232 were downregulated in intracellular *M. tb* 1458. Compared to extracellular BCG, 121 ncRNAs were upregulated and 335 downregulated in extracellular *M. tb* 1458 (Figure 2B).

**FIGURE 3:**
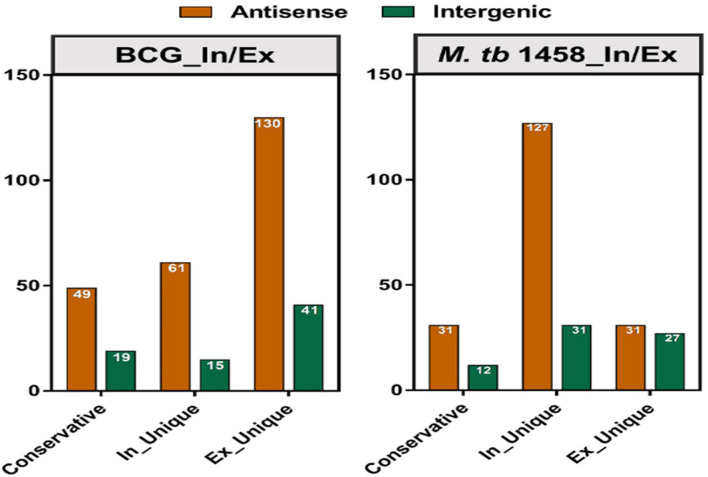
*Mycobacterium tuberculosis* 1458/BCG count distribution of differentially expressed ncRNAs in different types between intracellular and extracellular bacteria (| FC| > 1.5).

**FIGURE 4:**
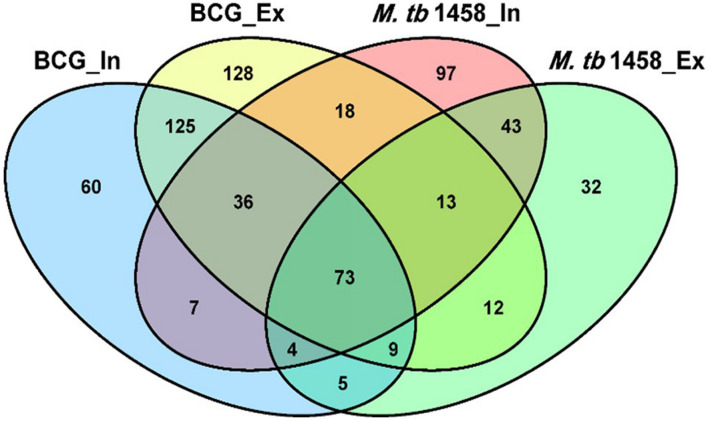
Venn diagram of the overlap after BCG and *Mycobacterium tuberculosis* 1458 ncRNA sequences are compared. In, intracellular; Ex, extracellular.

Among differentially expressed ncRNAs, 68 were conserved in BCG and 43 were conserved in *M. tb* 1458. There were more upregulated ncRNAs in extracellular BCG than *M. tb* 1458 (Figure 3). Compared to BCG and *M. tb* 1458, 73 were expressed in all groups, 18 ncRNAs were expressed in extracellular BCG and intracellular *M. tb* 1458, and only 5 were expressed in intracellular BCG and extracellular *M. tb* 1458 (Figure 4).

### Non-coding RNA Annotation

**FIGURE 5:**
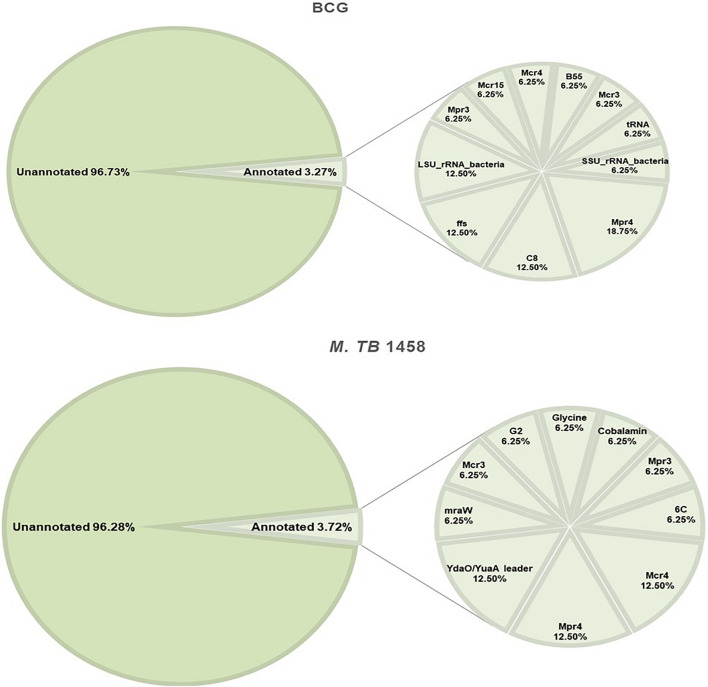
Annotation results of BCG and *Mycobacterium tuberculosis* 1458 ncRNAs by Rfam and BRSD sRNA databases.

Rfam and BRSD databases were used to annotate ncRNAs. Moreover, 96.73% in BCG and 96.28% in *M. tb* 1458 were novel ncRNAs (Figure 5). For the annotated ones, a very small part was rRNAs and tRNAs, and the others were reported sRNAs with regulatory functions, such as sRNA G2, C8, glycine, mraW, 6C, and ydaO-yuaA (Mandal et al., 2004; Weinberg et al., 2007, 2010; Kwon and Strobel, 2008; Arnvig and Young, 2009; Nelson et al., 2013; Mai et al., 2019). All annotated ncRNAs were removed; the remaining differentially expressed ncRNAs were used to predict the target and analyze the pathway enrichment.

IntaRNA, CopraRNA, and TargetRNA2 were used to predict the target genes. Genes predicted by all three tools were used for the following analysis. Some of the ncRNA target genes are listed in Table 1. In all nine listed ncRNAs, eight target genes were related to lipid metabolism, such as lipid proteins *lpqK*, *lspA*, and, *lprQ*, fatty acid metabolism related to the enzyme enyl-CoA hydrases *echA18*, *echA21*, *JTY_0827*, and acetyl-CoA dehydrogenases *fadE3*, *fadE16*, *fadD18*, *fadD28*, and *fadE29*.

**TABLE 1:** Important target genes of some differentially expressed ncRNAs.

**DEG ncRNAs**	**Targets ID**	**Target locus**	**Annotation**	**Hybrid energy**
ncBCG201	BQ2027_MB0405C	lpqK	Possible conserved lipoprotein	−67.7
	BQ2027_MB1706	fadE16	Possible acyl-CoA dehydrogenase	−61.9
	BQ2027_MB3504	PE31	PE family protein	−60.7
ncBCG343	BQ2027_MB1575C	PPE21	PPE family protein	−35.7
	BQ2027_MB3477C	eccc4	Esx-4 type VII secretion system protein. Probable membrane protein	−31.1
	BQ2027_MB1812	eccc5	Esx-5 type VII secretion system protein	−30.9
ncBCG356	BQ2027_MB3408	echA18	Probable enoyl-CoA hydratase (unsaturated acyl-CoA hydratase)	−52.3
	BQ2027_MB3918C	espg2	Esx-2 secretion-associated protein	−51.3
	BQ2027_MB3542C	fadD18	Probable fatty-acid-CoA ligase (fatty-acid-CoA synthetase)	−48.4
ncBCG427	JTY_RS06565	JTY_1269	Membrane protein	−20.7
	JTY_RS08105	lspA	Lipoprotein signal peptidase	−16.4
	JTY_RS04260	JTY_0827	Phosphodiesterase	−16.1
ncMTB104	Rv3543c	fadE29	Acyl-CoA dehydrogenase	−50.2
	Rv1942c	mazF5	Toxin	−46.7
	Rv0930	pstA1	Phosphate ABC transporter permease	−46.4
ncMTB208	Rv2941	fadD28	Long-chain-fatty-acid-AMP ligase	−44.5
	Rv0981	mprA	Two-component response regulator	−43.4
	Rv2053c	fxsA	Transmembrane protein	−42.0
ncMTB215	Rv3445c	esxU	ESAT-6 like protein	−29.4
	Rv1846c	blaI	Transcriptional repressor	−26.5
	Rv3881c	espB	ESX-1 secretion-associated protein	−26.4
ncMTB224	Rv3904c	esxE	ESAT-6 like protein	−53.0
	Rv0483	lprQ	Lipoprotein	−46.6
	Rv0915c	PPE14	PPE family protein	−45.5
ncMTB233	Rv3260c	whiB2	Transcriptional regulator	−45.5
	Rv3330	dacB1	Penicillin-binding protein	−46.2
	Rv3260c	whiB2	Transcriptional regulator	−44.6

**FIGURE 6:**
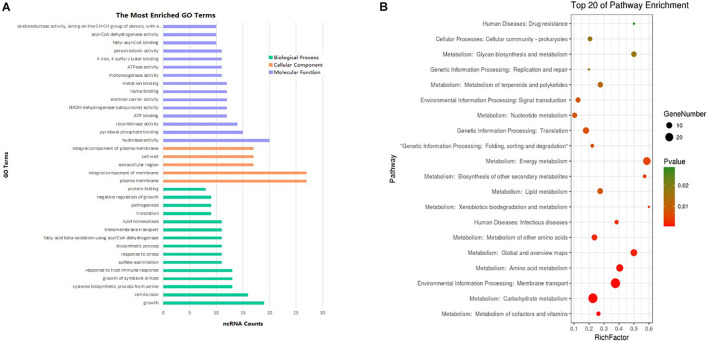
Enrichment analysis of differentially expressed ncRNAs (| FC| > 1.5). **(A)** GO pathways (*p* < 0.1) of the targets most enriched in biological process, cell component, and molecular function. **(B)** Top 20 KEGG enrichment results (*p* < 0.1) of the target genes.

The most prevalent GO enrichment analyses were biological processes and molecular functions, followed by cellular components (Figure 6A). Among the top 15 terms in biological processes, growth, pathogenesis, growth of symbiont in host, and transmembrane transport were all related to *Mycobacterium* infection and intracellular survival. Among the top 15 terms in molecular functions, fatty acyl-CoA binding and acyl-CoA dehydrogenase activity were strongly related to *Mycobacterium* fatty acid metabolism. ncRNAs in cellular components group were significantly lower than other two groups.

To analyze differentially expressed ncRNA target genes using the KEGG pathways, the top 20 were mainly related to bacterium metabolism, human diseases, genetic information processing, and environmental information processing (Figure 6B). The interaction network of pathways are presented in Supplementary Figures 1–3. Because carbohydrate supply energy for bodies, carbohydrate metabolism network was by no doubt the most complex one. Because almost all subgroups had ncRNAs, we decided not to focus on them. As lipids are one of the three major nutrients groups in life and are essential for bacterial survival, added to the fact that functional enzymes associated with fatty acid metabolism have an important impact on the virulence regulation of *M. tb*, this study mainly focused on lipid metabolism, both in synthesis as well as degradation. ncBCG314 was related to glycerophospholipid metabolism, glycerolipid metabolism, and fatty acid metabolism; ncBCG150 was related to glycerolipid metabolism, fatty acid metabolism, and fatty acid degradation; ncBCG428 and ncBCG356 were associated with fatty acid metabolism, fatty acid degradation, and synthesis and degradation of ketone bodies; ncBCG285 and ncBCG112 were related to glycerophospholipid metabolism, fatty acid metabolism, and synthesis and degradation of ketone bodies; ncBCG98, ncBCG236, ncBCG337, ncMTB248, and ncMTB233 were related to both glycerophospholipid and glycerolipid metabolism; and ncMTB329 was related to both synthesis and degradation of ketone bodies and glycerolipid metabolism (Supplementary Figure 1).

For sRNAs related to human diseases, ncBCG184, ncBCG285, ncBCG156, ncBCG341, ncBCG126, ncMTB64, ncMTB174, ncMTB165, ncMTB258, and ncMTB254 were related to TB. Among them, ncBCG285 and ncMTB165 were also related to biosynthesis, indicating that the two sRNAs participate in drug-resistant *Mycobacterium* infection (Supplementary Figure 2).

In genetic and environmental information processing pathways, the number of ncRNAs in the ribosomal pathway was the highest. Besides, pathways of bacterium environmental processing, including membrane transportation and two-component system, were also included, as well as membrane transportation pathway, including ABC transporter and bacterial secretion system. Many ncRNAs were cross-enriched to more than one pathway: ncBCG429 was related to mismatch repair, microbial metabolism in diverse environments, and two-component system; ncBCG34 was involved in microbial metabolism in diverse environments, two-component system, and sulfur relay system; ncBCG403 was related to two-component system, protein export, and bacterial secretion system; and ncBCG186 was related to sulfur relay system, protein export, and bacterial secretion system. In *M. tb* 1458, more ncRNAs participated in multiple pathways: ncMTB147 was related to protein export, bacterial secretion system, nucleotide excision repair, and quorum sensing; ncMTB258 was involved in protein export, bacterial secretion system, and quorum sensing; ncMTB206 was involved in nucleotide excision repair, mismatch repair, and sulfur relay system; and ncMTB211, ncMTB20, and ncMTB164 were related to both microbial metabolism in diverse environments and ribosome (Supplementary Figure 3).

### Identification of Stress Regulation-Related Non-coding RNAs

**FIGURE 7:**
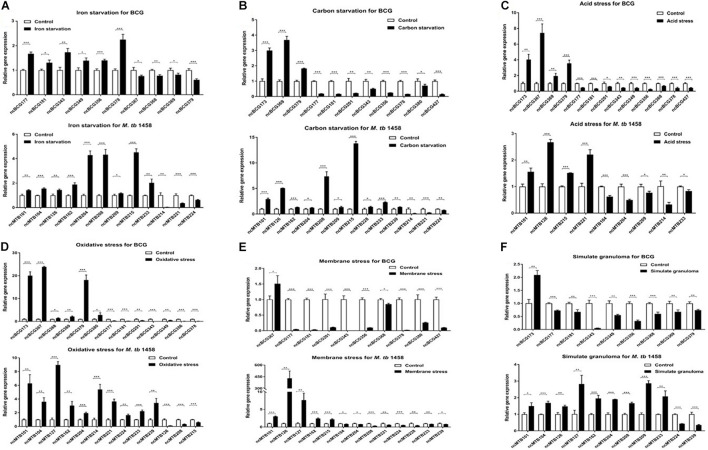
Detection of BCG and *M. tb* 1458 DEG ncRNAs under different stress culture model. **(A)** Iron starvation model; **(B)** carbon starvation model; **(C)** acid stress model; **(D)** oxidative stress model; **(E)** membrane stress model; **(F)** simulate granuloma stress model. The data used the mean ± SD of three replicate samples, *t*-test was used for statistical analysis, *p < 0.05, **p < 0.01, and ***p < 0.001.

Seventy-five differentially expressed ncRNAs (including 37 BCG ncRNAs and 38 *M. tb* 1458 ncRNAs) with potential biological functions predicted by biological information were selected and tested using relative RT-PCR in six stress culture models. Thirteen BCG ncRNAs and 14 *M. tb* 1458 ncRNAs were significantly expressed in the stress environment. Differentially expressed ncRNAs in different stress culture models are presented in Figure 7. ncBCG177, ncBCG181, ncBCG343, ncBCG356, ncMTB101, ncMTB126, ncMTB204, and ncMTB233 were differentially expressed in all six stress environments. ncBCG368, ncBCG369, ncBCG378, ncBCG379, ncMTB104, ncMTB162, ncMTB208, ncMTB215, ncMTB221, and ncMTB224 were differentially expressed in five stress environments. ncBCG173, ncBCG201, ncMTB209, ncBCG349, ncBCG367, ncMTB214, and ncMTB239 were differentially expressed in four stress environments. ncBCG390, ncBCG427, and ncMTB127 were differentially expressed in three stress environments. ncMTB228 was differentially expressed in two stress environments.

For those ncRNAs, ncMTB162 was an antisense sRNA; ncBCG343, ncBCG378, and ncMTB208 were adjacent to the 5′-untranslated region (UTR) of downstream mRNA, and the others were located in gene intergenic regions (Supplementary Figure 4). Secondary structure prediction results showed that the loop of ncBCG177, ncBCG181, ncBCG201, ncBCG343, ncBCG356, ncBCG378, ncBCG427, ncMTB104, ncMTB162, ncMTB204, ncMTB208, ncMTB215, ncMTB224, and ncMTB233 was between 4 and 14 bp and had high GC content, indicating that these sRNAs can bind to target mRNA more precisely and firmly (Supplementary Figure 5).

### Phenotypic Testing of Recombinant *Mycobacterium smegmatis*

**FIGURE 8:**
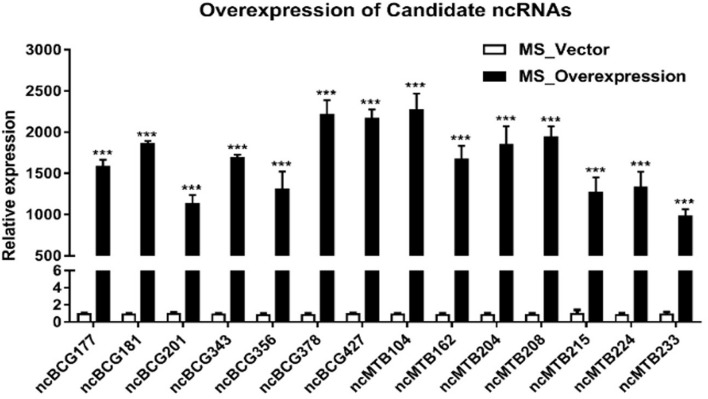
Expression of candidate sRNAs in each overexpressing MS strain. Data are the mean ± SD of three replicate samples. ***p < 0.001 (*t*-test).

**FIGURE 9:**
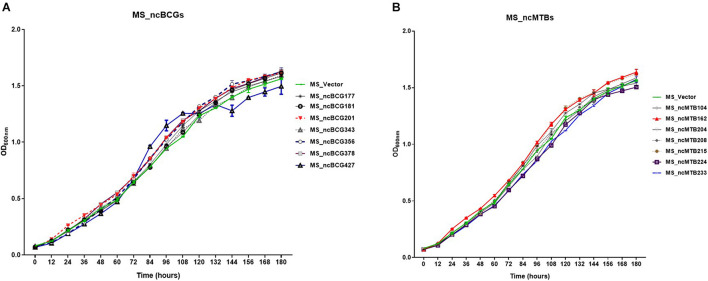
Growth curve of candidate sRNAs overexpressing *Mycobacterium smegmatis* MC^2^155. **(A)** ncBCG sRNA overexpressing *M. smegmatis* MC^2^155. **(B)** ncMTB sRNA overexpressing *M. smegmatis* MC^2^155.

Fourteen sRNAs (including 7 ncBCG RNAs and 7 ncMTB RNAs) with less than 100 bp were successfully overexpressed in *M. smegmatis* (Figure 8). The recombinant MS Vector was used as a control. For the growth curves, MS_ncBCG201 showed a significantly greater growth speed since 24 h (*p* < 0.05), especially significantly higher from 94 h (*p* < 0.0001). MS_ncBCG427 showed a significant increase in growth since 84 h (*p* < 0.0001) but quickly entered the plateau phase at 120 h and grew slowly since 144 h (*p* < 0.0001). MS_ncBCG378 and MS_ncBCG356 also showed a higher growth speed since 60 and 84 h, respectively (*p* < 0.001; Figure 9A). For recombinant *M. smegmatis* of ncMTB sRNAs, MS_ncMTB162 grew significantly quicker from 24 h (*p* < 0.0001). MS_ncMTB233 and MS_ncMTB224 showed a similar trend and grew slowly since 48 h (*p* < 0.001 and *p* < 0.01). MS_ncMTB215 grew significantly quickly since 84 h and lasted for a long time (*p* < 0.0001; Figure 9B).

**FIGURE 10:**
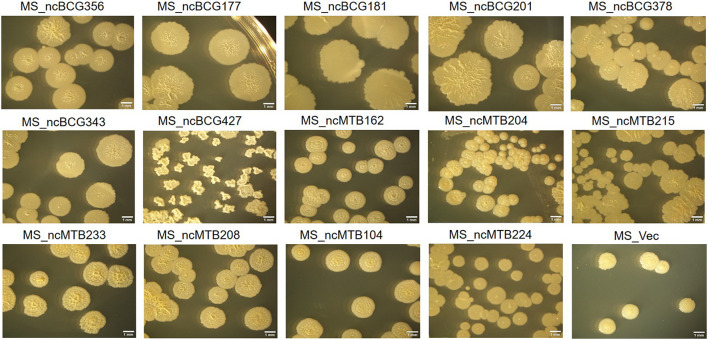
Single-colony morphology of candidate sRNAs overexpressing *Mycobacterium smegmatis* MC^2^155.

**FIGURE 11:**
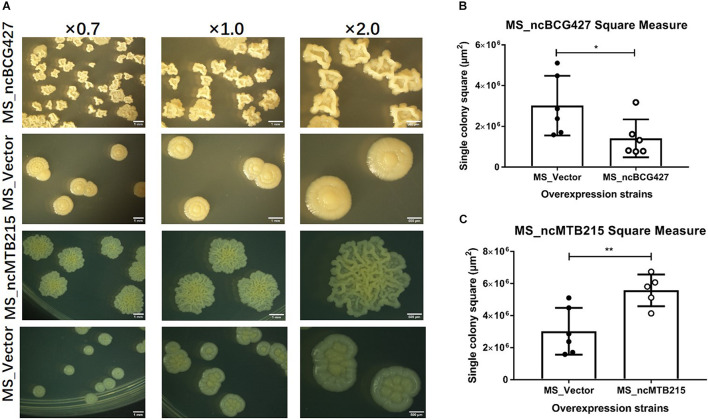
Single-colony morphology and square measure of overexpressing *Mycobacterium smegmatis* MC^2^ 155: MS_ncBCG427 and MS_ncMTB215. **(A)** Single-colony morphology of different strains. **(B)** Square of MS_ncBCG427. **(C)** Square of MS_ncMTB215. *p < 0.05; **p < 0.01 (*t*-test).

Single-colony morphology was also tested. The recombinant MS_Vector showed a smooth and moist morphology. MS_ncBCG356, MS_ncBCG177, MS_ncBCG181, MS_ncBCG201, MS_ncBCG378, MS_ncBCG343, and MS_ncMTB224 showed a thinner colony. Except for MS_ncMTB224, the other six strains had a significantly larger colony than control (Figure 10). The most different one was MS_ncBCG427, which showed a significantly smaller and rough colony (Figures 11A,B), followed by MS_ncBCG215, which was larger but also rough (Figures 11A,C). These two strains had very obviously folds, whose ridge was obviously higher than control, indicating that sRNA ncBCG427 and ncMTB215 may affect bacterial growth by regulating the cell wall components.

### Biofilm-Forming Ability of Recombinant *Mycobacterium smegmatis*

**FIGURE 12:**
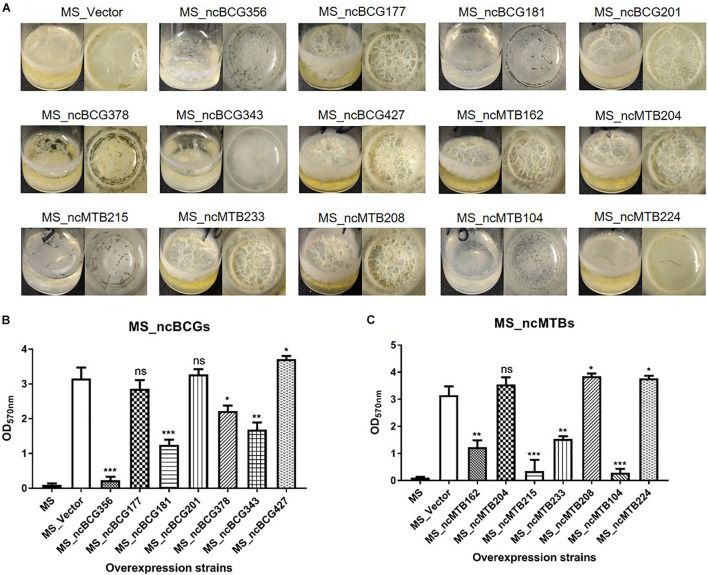
Biofilm-forming ability of candidate sRNA overexpressing strains. **(A)** Biofilm morphology. **(B)** Crystal violet staining results of MS_ncBCGs and **(C)** MS_ncMTBs compared to MSJ_Vector. *p < 0.05; **p < 0.01; ***p < 0.001 (*t*-test).

The biofilm differences between the sRNA overexpressed strain and MS_Vector were compared. Except for MS_ncBCG177, MS_ncBCG201, and MS_ncMTB204, the other recombinant strains had significantly different biofilm-forming abilities compared to MS_Vector. The biofilms of MS_ncBCG356, MS_ncBCG181, MS_ncBCG378, MS_ncBCG343, MS_ncMTB215, and MS_ncMTB104 were sparse and easy to be broken under shaking. Although MS_ncMTB162 and MS_ncMTB233 had lower biofilm-forming abilities, they were still robust and not fragile. MS_ncBCG427, MS_ncMTB208, and MS_ncMTB224 had higher biofilm-forming abilities compared to MS_Vector (Figure 12).

### ncBCG427 Target Gene Prediction and Identification in Bacillus Calmette-Guérin

**FIGURE 13:**
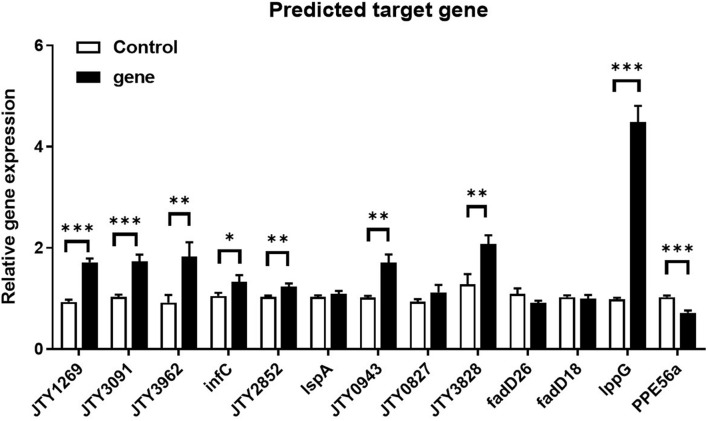
Differential expression of ncBCG427 predicted target genes in BCG. ^∗^*p* < 0.05; ^∗∗^*p* < 0.01; ^*⁣*⁣**^p < 0.001 (*t*-test).

Principle component analysis (PCA) and snapshots of ncBCG427 from intracellular and extracellular fractions were represented in Supplementary Figure 6. A total of 46 target genes were enriched in biological process, 38 related in cellular component and 69 associated with molecular function (Supplementary Table 4). RT-PCR was used to identify target gene expression. Nine genes (*JTY1269*, *JTY3091*, *JTY3962*, *infC*, *JTY2852*, *JTY0943*, *JTY3828*, *lppG*, and *PPE56a*) were upregulated (Figure 13) in BCG_ncBCG427 strain cultured in a 7H9 medium with 50 μg/ml kanamycin in 37°C, gene expression levels in wild type BCG strain were used as control. Among those nine genes, *infC* was a translation initiation factor associated with biofilm formation, *JTY2852* was a bifunctional oligoribonuclease, *JTY0943* was a IS607 family transposase, *JTY_3828* was a SAM-dependent methyltransferase, *lppG* was a lipoprotein, *PPE56a* belongs to the PPE family with unknown function.

## Discussion

The binding of sRNA to its target not only prevents ribosome binding but also initiates the cleavage of RNA-RNA interaction. In *Staphylococcus aureus*, for example, sRNA controls the expression of several pathogenesis-related genes binding to the target RNA (Ostrik et al., 2021). An initial analysis of the *M. tb* transcriptome by RNA-seq revealed the presence of at least 15 sRNAs (Westermann, 2018). Currently, many more regulatory sRNAs have been identified in *Mycobacterium* using saturated transposon mutagenesis (Budell et al., 2020). More recent studies have evidenced the importance of sRNA in pathogenesis. Therefore, sRNAs represent a regulatory mechanism that provides an adaptive response to fast-changing environments and potentially are quite important in slow-growing organisms.

This study attempted to identify regulatory mechanisms in the human pathogen *M. tb* and the animal pathogen *M. bovis*. By exposing both microorganisms to conditions encountered in the host, many new sRNA sequences were identified.

### RNA-Seq Comparison Between Extracellular and Intracellular Samples of Bacillus Calmette-Guérin and *Mycobacterium tuberculosis*

Bacillus Calmette-Guérin and *M. tb* 1458 to infect THP-1 cells were used to characterize ncRNA differences both inside and outside the infected cells. A previous study demonstrated that *M. tb* 1458 was capable of infecting macrophages more efficiently and has an enhanced virulence compared to *M. bovis* BCG (Xiong et al., 2017; Zhang et al., 2018). ncRNAs obtained from extracellular BCG were more abundant than those obtained from the intracellular environment. In comparison, there were less ncRNAs in extracellular than intracellular *M. tb*. This observation may be related to the fact that virulent *M. tb* 1458 has higher macrophage uptake. For all annotated ncRNAs, about 20% were rRNA and tRNA, which are essential for ribosome and translation (Ravishankar et al., 2016), and generally are not involved in the post-transcriptional level of regulation. Mcr3 (Mpr7) and Mpr4 were expressed in both BCG and *M. tb* 1458 in this study. Mcr3, also reported as a conserved sRNA in *M. smegmatis*, may be involved in regulating functions in different mycobacteria (DiChiara et al., 2010). C8, a 4.5S RNA, is an antisense sRNA known to be trimmed off the first 90 nucleotides and is highly conserved in mycobacteria and more distantly related bacteria, such as Rhodococcus, Corynebacteria, and Nocardia (Arnvig and Young, 2009). B55 and G2 are two sRNAs reportedly conserved in *M. tb* complex. B55 may be part of the 3′-UTR of the Rv0609A mRNA rather than being a sRNA. G2 is a *trans-*encoded sRNA with more than one 5′-end and may act as a possible SigC promoter upstream of one of the 5′-ends that can downregulate the growth of *Mycobacterium* (Arnvig and Young, 2009). Glycine riboswitch was only reported in *Bacillus subtilis*, regarded as a unique *cis-*acting mRNA element that contains two tandem homologous glycine-binding domains and acts on a single-expression platform to regulate gene expression in response to glycine (Babina et al., 2017). Riboswitching-binding metabolites have been successfully used to inhibit the growth of non-pathogenic *B. subtilis in vitro* (Ritchey et al., 2020). 6C is a conserved sRNA in high GC Gram-positive bacteria, including *Mycobacterium*; it has many cellular functions *via* binding to multiple mRNA targets through the C-rich loops in *Mycobacterium* and regulates DNA replication and protein secretion (Mai et al., 2019). YadO is a riboswitch that regulates the resistance to enzyme-mediated degradation (Meehan et al., 2016).

This study showed that many sRNAs expressed intracellularly at the time point examined have functions that are not completed understood, and future studies will have to address the knowledge gap. It is also important to consider that cell-to-cell heterogenicity in RNA expression may occur.

### Biofilm-Forming Ability

Biofilm plays an important role in *Mycobacterium* pathogenicity and also drug resistance. Biofilm-forming ability influences bacterial existence in adverse environments. In mycobacteria, cell surface molecules, especially many lipids, are associated with biofilm formation, such as glycopeptidolipids, poly-a-L-glutamine, mycolic acid, and PPE family proteins (Xiang et al., 2014; Crotta Asis et al., 2021). *M. tb* and *M. bovis* form cords and biofilm inside the granulomatous lesion as well as intracellularly. The role of biofilm in these conditions is not well-understood and may be related to the absence of nutrients, although, inside the cells, the synthesis of trehalose-6-6-dimycolate (TDM or cord factor) may be reduced due to the shift in metabolism related to the abundance of glucose, leading to the synthesis of glucose monoglycolate instead of TDM (Hunter et al., 2006).

Here, differentially expressed sRNAs ncBCG201 (RPKM fold of extracellular/intracellular was 2.24), ncBCG343 (unique expressed intracellularly) and ncMTB224 (unique expressed in extracellular *M. tb* 1458) were predicted targets to the PE/PPE family genes. A previous study reported that PE/PPE genes are mainly found in virulent mycobacteria (Li et al., 2019), which agree with the differentially expressed results, as PE/PPE proteins are important for *Mycobacterium* growth and could be differentially expressed under a variety of conditions (Li et al., 2019), suggesting that ncBCG201, ncBCG343, and ncMTB224 might participate in the pathogenesis and growth of the bacterium.

The recombinant strain of the three sRNAs all showed a significant change in the growth rate (not obviously for ncBCG343), small colonies, and different biofilm-forming abilities, which were all associated with the virulence of the bacterium (Ramsugit and Pillay, 2014; Cardona, 2018), confirming that ncBCG201, ncBCG343, and ncMTB224 have potential regulating pathogenesis ability. In addition, all three RNAs changed the drug resistance ability, indicating that they may be also targeted in some genes associated with drug resistance.

### Lipid Metabolism Regulation Ability

Lipids constitute a large percentage of the mycobacterial outer structure. Many virulent mycobacteria, such *Mycobacteria avium* subsp. *paratuberculosis*, change the lipids of the cell wall after infection (Alonso-Hearn et al., 2010). This study showed that lipid metabolism is strongly associated with many target genes of differentially expressed sRNAs, such as *lpqK*, *lspA*, *lprQ*, enyl-CoA hydrases *echA18*, *echA21* and *JTY_0827*, and acyl-CoA dehydrogenases fadE3, fadE16, fadD18, fadD28, and fadD29.

The composition and organization of the mycobacterial cell envelope is very complex, a distinctive feature of the *Mycobacterium* genus. The cell envelope plays multiple roles in *Mycobacterium* infection, including the modulation of the phagosome maturation and granuloma biogenesis, and is involved in the ability of the bacteria to adapt to all kinds of environmental stress (Gago et al., 2018). Lipid metabolism, especially fatty acid metabolism, is essential in the *Mycobacterium* life cycle, and can affect bacterial virulence and drug tolerance during infection (Nazarova et al., 2017).

*Mycobacterium tuberculosis* encodes five type VII secretion systems (ESX-1–ESX-5), whose genes are arranged in highly conserved clusters. Here, esxU and espB (ncMTB215), and esxE (ncMTB224), associated with ESX-1, can modulate necrosis, NOD2 signaling, type I interferon production, and autophagy (Wong, 2017). Eccc5 (ncBCG343), an Esx-5 type VII secretion system protein, is essential for growth and participates on the cell surface and cell envelope fraction transportation (Ates et al., 2015). espg2 is a Esx-2 secretion-associated protein, although its function is unclear.

As target genes of differentially expressed ncRNAs mainly participate in the biological process, not cell component, although they are multiple functions, the GO enrichment analysis still discovered that the top function was associated with lipid or membrane. This information was also confirmed by the KEGG analysis.

### Ability to Adapt to the Environment

Tuberculosis is an old disease. For thousands of years, pathogens evolved in many ways to survive within the host and adapt to the ever-changing environment, both extracellular and intracellular. In different nutritionally defined environments, such as lack of carbon and nitrogen sources, and low tension of oxygen environment, the lipid composition of the envelope will be changed, resulting in the alteration of growth rate, metabolic activity, and antibiotic resistance (Gago et al., 2018). Under different stresses and environments, almost all sRNAs recombinantly expressed in *M. smegmatis* had some sort of change in bacterial adaptation. More specifically, ncBCG177, ncBCG181, ncBCG343, ncBCG356, ncMTB204, and ncMTB233 not only changed the expression level in all six stress models but also altered bacterial growth rate and colony morphology, suggesting that expressed sRNAs encoded the ability of the bacterium to adapt to adverse environments. Among the strains, *M. smegmatis* expressing ncBCG427 had the most different morphology. Its expression had effects of biofilm robustness, antibiotic resistance, and improved ability of immune escape, changing pathogen virulence (Rastogi et al., 2017; Wang et al., 2019).

## Conclusion

Taken together, 490 ncRNAs in BCG and 349 ncRNAs in *M. tb* 1458 were identified by RNA-seq. In a subset of differentially expressed sRNAs in multiple stress conditions, many were associated with lipid metabolism. Among them, ncBCG427 was significantly downregulated when BCG entered into macrophages and was associated with increased biofilm formation. Then, the reduction of virulence possibility depends on regulating lipid metabolism. ncBCG427, which is significantly downregulated when *M. bovis* BCG is ingested by macrophages, is associated with biofilm formation in *Mycobacterium* and reduces the virulence-related phenotype that depends on lipid metabolism.

Previous studies have indicated the role of lipids in *M. tb* virulence and supported the observations that lipids are constantly released from the bacterium, very likely indicating their crucial role in pathogen survival. Understanding the regulation of lipid synthesis and release will provide important information about future drug targets.

## Data Availability Statement

The authors acknowledge that the data presented in this study are deposited in the SRA database repository, accession number PRJNA755755.

## Author Contributions

AG and YC contributed to the conception and design of the study. WZ and KZ carried out the experiment and wrote sections of the manuscript. HL and LB performed the statistical analysis. YC wrote the first draft of the manuscript. All authors contributed to manuscript revision, read, and approved the submitted version.

## Conflict of Interest

The authors declare that the research was conducted in the absence of any commercial or financial relationships that could be construed as a potential conflict of interest.

## Publisher’s Note

All claims expressed in this article are solely those of the authors and do not necessarily represent those of their affiliated organizations, or those of the publisher, the editors and the reviewers. Any product that may be evaluated in this article, or claim that may be made by its manufacturer, is not guaranteed or endorsed by the publisher.

## Footnotes

1
http://rfam.xfam.org/

2
http://kwanlab.bio.cuhk.edu.hk/BSRD/

3
https://david.ncifcrf.gov/home.jsp

4
http://kobas.cbi.pku.edu.cn/

5
http://rna.informatik.uni-freiburg.de/IntaRNA/Input.jsp

6
http://rna.informatik.uni-freiburg.de/CopraRNA/Input.jsp

7
http://cs.wellesley.edu/∼btjaden/TargetRNA2/index.html
