# Supplementary material for: Small RNA Profiling in Mycobacterium Provides Insights Into Stress Adaptability
Source: Front Microbiol. 2021 Nov 4;12:752537. doi: 10.3389/fmicb.2021.752537 (PMC8600241; doi:10.3389/fmicb.2021.752537)
Supplement: Supplementary file 1 [file Table_1.DOCX]

**STable 1 Primers that were used for RT-PCR in this study**

| Name | Primers | Tm (°C) | Size (bp) |
| --- | --- | --- | --- |
| ncRv0710 | F: 5′- ctggcggctcgtcgagat -3′  R: 5′- caaaagcccccgcacgcc -3′ | 61 | 91 |
| ncRv11429 | F: 5′- cgtcaaacaacccgacca -3′  R: 5′- tgggtatacgccgggtcc -3′ | 62 | 66 |
| ncRv11803 | F: 5′- gcggacccgctatggggt -3′  R: 5′- gaccccgcttggggtgac -3′ | 60 | 74 |
| ncRv11846 | F: 5′- tcgtcgagccacaacgac -3′  R: 5′- caccaacgggcgagcccc -3′ | 63 | 106 |
| ncRv12497c | F: 5′- agcgccagtgttgcggcg -3′  R: 5′- tctcctcagctcgcgatc -3′ | 56 | 174 |
| ncRv2796c | F: 5′- ccctcgccgaccccgtag -3′  R: 5′- caaaagcccccatttcgggcc -3′ | 60 | 173 |
| ncRv2993c | F: 5′- atgcgtatcggtcgaatc -3′  R: 5′- gtcggcgtaccgaacggg -3′ | 60 | 84 |
| ncRv3402c | F: 5′- ccccggatgacgttgccc -3′  R: 5′- ctgggggcaccatccgct -3′ | 60 | 122 |
| ncRv13803c | F: 5′- cacaacatctagcggcggtg -3′  R: 5′- aacatctagcggcggcga -3′ | 61 | 149 |
| ncRv13907 | F: 5′- ccttgggtgcggtcgcgt -3′  R: 5′- ggagcggggcggtgcagg -3′ | 62 | 48 |
| ncRv12659 | F: 5′- gtcgcctggtcagaggct -3′  R: 5′- gaggaacctttcggtggt -3′ | 60 | 130 |
| ncBCG356 | F: 5′- AGCCGCTTCGTCACATGCTGT -3′ | 63 | 97 |
| ncBCG177 | F: 5′- AGCCGCTTCGTCACATGCTGT -3′ | 60 | 73 |
| ncBCG181 | F: 5′- GGTGAACGACACCAGATCGG -3′ | 60 | 71 |
| ncBCG201 | F: 5′- ATGCTGGGCGCCCGGGTCGG -3′ | 61 | 88 |
| ncBCG378 | F: 5′- ACGCAATGCTCCCGTCATATC -3′ | 62 | 77 |
| ncBCG343 | F: 5′- TCATTCGTACACATTGAGAAT -3′ | 60 | 67 |
| ncBCG427 | F: 5′- ATGCACGACGACCCTCGCCTC -3′ | 63 | 80 |
| ncMTB104 | F: 5′- GCTCGAAGGAGGTTGGCACGT -3′ | 65 | 68 |
| ncMTB162 | F: 5′- GACGCGGGCCAGGACCTTGG -3′ | 60 | 96 |
| ncMTB215 | F: 5′- GCAAGCGTGTTGTTTGAGAAC -3′ | 61 | 80 |
| nc MTB208 | F: 5′- GAGGGGAAGCCGCTGGGACT -3′ | 62 | 66 |
| ncMTB233 | F: 5′- TCGTCCGTGCAGCAGTGAACG -3′ | 60 | 83 |
| ncMTB224 | F: 5′- AAGGTGAAGATACCCGAGAAC -3′ | 63 | 86 |
| All-in-one | R: 5′- GCTGTCAACGATACGCTACGTAAC -3′ | 64 |  |
| sigA | F: 5′- ACCCACCGAAAAGGACAAGG -3′  R: 5′-CTGTTTGAGGTAGGCGCGAA -3′ | 60 | 132 |

**STable 2 Primers that were used in target gene validation**

| Gene name | Primers | Tm (°C) | Size (bp) |
| --- | --- | --- | --- |
| JTY_1269 | F: 5′- ACAGGTTCCCGGTTCAACAC -3′  R: 5′-GGGAACTGCTGCTCGGATAG -3′ | 59 | 153 |
| JTY_3091 | F: 5′-CCTGTGGCAAAAGCTCATGG-3′  R: 5′-CCCTACGCACCAGCTGAAAT-3′ | 57 | 191 |
| JTY_3962 | F: 5′-AGCTTTGTCGAATCCCCGTT-3′  R: 5′-ACAACTACTCGTTGGCCGAG-3′ | 57 | 196 |
| infC | F: 5′-CGAATCCCGCAGAAACCAAC-3′  R: 5′-GCTCACGTCCACGGAACATA-3′ | 57 | 158 |
| JTY_2852 | F: 5′-CGGGTCAATGAAATTCGCGG-3′  R: 5′-CGCGATGTCGATTTGGTTGT-3′ | 57 | 150 |
| lspA | F: 5′-ACCTGGGTTTTGACGCTGAT-3′  R: 5′-CAAGAAATCGACGACGTGCC-3′ | 57 | 183 |
| JTY_0943 | F: 5′-GGGTGAATCGACATACCGCT-3′  R: 5′-TATCATGGCTTGACACCCGC-3′ | 57 | 156 |
| JTY_0827 | F: 5′-GTTGCATCATCCGCCGATTC-3′  R: 5′-CAGGTCCTGGGTGTAGCAAG-3′ | 59 | 199 |
| JTY_3828 | F: 5′-CGCAACGTGGCTGATTTGAA-3′  R: 5′-ACCGCGTTTTTCGACGAGTA-3′ | 55 | 173 |
| fadD26 | F: 5′-CGTGGTAGGCGATGTAACGA-3′  R: 5′-CGTCGATCCGGACGTGTATT-3′ | 57 | 163 |
| fadD18 | F: 5′-AGTAGCCGACGGGAATGTTG-3′  R: 5′-TGTGCCGGCTTTCTGGTAAT-3′ | 57 | 138 |
| lppG | F: 5′-TGGACCATTCTGTCTCAGCG-3′  R: 5′-CCCATTCGTTGTGCTGGTTG-3′ | 57 | 145 |
| PPE56a | F: 5′-CCGGTCCCCGAATTGAAGAA-3′  R: 5′-GTGTGGGCAACATCGGTTTC-3′ | 57 | 157 |

**STable 3 General information of total reads**

| Sample | Total reads | Total mapped reads | Uniq mapped reads | multiple mapped reads |
| --- | --- | --- | --- | --- |
| MTB24_in | 4901464 | 1082395(22.08%) | 988915(91.36%) | 93480(8.64%) |
| MTB24_out | 3502598 | 770272(21.99%) | 705598(91.60%) | 64674(8.40%) |
| MTB6_in | 4961872 | 1064835(21.46%) | 973006(91.38%) | 91829(8.62%) |
| MTB6_out | 4866369 | 992473(20.39%) | 905196(91.21%) | 87277(8.79%) |
| BCG24_in | 5160322 | 1139286(22.08%) | 1069077(93.84%) | 70209(6.16%) |
| BCG24_in | 6789394 | 1524223(22.45%) | 1417271(92.98%) | 106952(7.02%) |
| BCG6_in | 7224959 | 1673832(23.17%) | 1549162(92.55%) | 124670(7.45%) |
| BCG6_out | 7062495 | 1655866(23.45%) | 1521143(91.86%) | 134723(8.14%) |

**STable 4 Important target genes of sRNA ncBCG427**

| Locus tag | Gene | Annotation |
| --- | --- | --- |
| jty_rs06565 | JTY_1269 | membrane protein |
| jty_rs15920 | JTY_3091 | Derived by automated computational analysis using gene prediction method: Protein Homology. |
| jty_rs20315 | JTY_3962 | Derived by automated computational analysis using gene prediction method: Protein Homology. |
| jty_rs08545 | infC | translation initiation factor IF-3 |
| jty_rs07270 |  | Derived by automated computational analysis using gene prediction method: Protein Homology. |
| jty_rs14675 | JTY_2852 | bifunctional oligoribonuclease/PAP phosphatase NrnA |
| jty_rs08105 | lspA | lipoprotein signal peptidase |
| jty_rs04885 | JTY_0943 | IS607 family transposase IS1535 |
| jty_rs04260 | JTY_0827 | phosphodiesterase |
| jty_rs19630 | JTY_3828 | SAM-dependent methyltransferase |
| jty_rs12965 | folC | 3-methyl-2-oxobutanoate dehydrogenase subunit alpha |
| jty_rs14270 | hsdS | restriction endonuclease subunit S |
| jty_rs12670 | rplU | 50S ribosomal protein L21 |
| jty_rs07475 | rpe | ribulose-phosphate 3-epimerase |
| jty_rs12070 | JTY_2340 | energy-coupling factor transporter transmembrane protein EcfT |
| jty_rs14785 | JTY_2847 | 3-oxoacyl-ACP reductase |
| jty_rs15035 | trmD | tRNA (guanosine(37)-N1)-methyltransferase TrmD |
| jty_rs16740 | JTY_3250 | GNAT family N-acetyltransferase |
| jty_rs15160 | fadD26 | long-chain-fatty-acid--AMP ligase FadD26 |
| jty_rs15365 | JTY_2985 | Derived by automated computational analysis using gene prediction method: Protein Homology. |
| jty_rs16840 | mtrA | DNA-binding response regulator |
| jty_rs18430 |  | Derived by automated computational analysis using gene prediction method: Protein Homology. |
| jty_rs01965 | JTY_0383 | xanthine dehydrogenase family protein subunit M |
| jty_rs13085 | lppS | transpeptidase |
| jty_rs03285 | JTY_0642 | prevent-host-death family protein |
| jty_rs07640 | secG | protein-export membrane protein SecG |
| jty_rs13910 | JTY_2696 | class I SAM-dependent RNA methyltransferase |
| jty_rs13875 | dxs1 | 1-deoxy-D-xylulose-5-phosphate synthase |
| jty_rs00110 | ppp | serine/threonine protein phosphatase |
| jty_rs12330 | mbtB | non-ribosomal peptide synthetase |
| jty_rs19060 | JTY_3718 | conjugal transfer protein |
| jty_rs11220 | JTY_2177 | division/cell wall cluster transcriptional repressor MraZ |
| jty_rs01525 | esxG | type VII secretion system protein EsxS |
| jty_rs05710 | echA9 | enoyl-CoA hydratase/isomerase family protein |
| jty_rs04025 | JTY_0781 | HIT family protein |
| jty_rs15210 | mas | mycocerosic acid synthase |
| jty_rs06450 | JTY_1246 | DUF3117 domain-containing protein |
| jty_rs02570 | regX3 | DNA-binding response regulator |
| jty_rs09290 | PPE25 | type VII secretion system ESX-5 target PPE25 |
| jty_rs19285 | JTY_3761 | ergothioneine biosynthesis PLP-dependent enzyme EgtE |
| jty_rs12530 | JTY_2427 | Derived by automated computational analysis using gene prediction method: Protein Homology. |
| jty_rs00430 | JTY_0081 | Derived by automated computational analysis using gene prediction method: Protein Homology. |
| jty_rs06840 | JTY_1325 | Derived by automated computational analysis using gene prediction method: Protein Homology. |
| jty_rs11900 | JTY_2307 | Derived by automated computational analysis using gene prediction method: Protein Homology. |
| jty_rs18040 | JTY_3514 | type VII secretion system ESX-4 subunit EccD4 |
| jty_rs14505 | JTY_2817 | Derived by automated computational analysis using gene prediction method: Protein Homology. |
| jty_rs02080 | JTY_0405 | Derived by automated computational analysis using gene prediction method: Protein Homology. |
| jty_rs00620 | JTY_0119 | sugar kinase |
| jty_rs13465 | PE_PGRS44 | PE family protein |
| jty_rs18295 | fdxD | ferredoxin |
| jty_rs02650 | JTY_0517 | Derived by automated computational analysis using gene prediction method: Protein Homology. |
| jty_rs07365 | PPE20 | PPE family protein |
| jty_rs04920 | pstS3 | phosphate-binding protein PstS |
| jty_rs01600 | JTY_0312 | TetR/AcrR family transcriptional regulator |
| jty_rs02780 | JTY_0543 | MinD/ParA family protein |
| jty_rs01030 | JTY_0198 | Derived by automated computational analysis using gene prediction method: Protein Homology. |
| jty_rs09705 | JTY_1888 | Derived by automated computational analysis using gene prediction method: Protein Homology. |
| jty_rs19245 | JTY_3753 | DUF58 domain-containing protein |
| jty_rs03620 | fabG | SDR family mycofactocin-dependent oxidoreductase |
| jty_rs18260 | mce4D | virulence factor Mce family protein |
| jty_rs18340 | fadD18 | fatty-acid--CoA ligase |
| jty_rs03500 | JTY_0683 | antitoxin |
| jty_rs07245 | JTY_1398 | Derived by automated computational analysis using gene prediction method: Protein Homology. |
| jty_rs08175 | frdC | fumarate reductase subunit C |
| jty_rs06825 | JTY_1322 | Derived by automated computational analysis using gene prediction method: Protein Homology. |
| jty_rs01095 | JTY_0211 | AI-2E family transporter |
| jty_rs04080 | JTY_0792 | oxidoreductase |
| jty_rs07335 | pyrC | dihydroorotase |
| jty_rs20170 | JTY_3930 | type VII secretion system ESX-1 associated protein EspF |
| jty_rs14350 | JTY_2787 | oxidoreductase |
| jty_rs00685 | JTY_0131 | maltokinase |
| jty_rs06780 | JTY_1313 | oxidoreductase |
| jty_rs13035 | orn | oligoribonuclease |
| jty_rs20405 | JTY_3981 | membrane protein insertase YidC |
| jty_rs11085 | JTY_2152 | Derived by automated computational analysis using gene prediction method: Protein Homology. |
| jty_rs05080 | JTY_0983 | Derived by automated computational analysis using gene prediction method: Protein Homology. |
| jty_rs04685 | JTY_0907 | diiron oxygenase |
| jty_rs07350 | carB | carbamoyl phosphate synthase large subunit |
| jty_rs14190 | JTY_2755 | transcriptional regulator |
| jty_rs16665 | JTY_3235 | ParA family protein |
| jty_rs11300 | JTY_2192 | Derived by automated computational analysis using gene prediction method: Protein Homology. |
| jty_rs09545 | glcB | malate synthase G |
| jty_rs13205 | aroF | chorismate synthase |
| jty_rs14745 | JTY_2866 | GCN5 family N-acetyltransferase |
| jty_rs12075 | JTY_2341 | ABC transporter ATP-binding protein |
| jty_rs14800 | glnA4 | glutamine synthetase |
| jty_rs06965 | JTY_1347 | DUF2550 domain-containing protein |
| jty_rs12980 | fadE19 | acyl-CoA dehydrogenase |
| jty_rs18015 | rplM | 50S ribosomal protein L13 |
| jty_rs01045 | JTY_0201 | DNA-binding response regulator |
| jty_rs01205 | JTY_0233 | DUF3068 domain-containing protein |
| jty_rs00900 | yrbE1A | membrane protein |
| jty_rs17885 | JTY_3483 | anti-sigma-D factor RsdA |
| jty_rs17510 | trpS | tryptophan--tRNA ligase |
| jty_rs13945 | JTY_2703 | DUF3710 domain-containing protein |
| jty_rs03400 | mmaA2 | cyclopropane mycolic acid synthase |
| jty_rs16885 | manA | mannose-6-phosphate isomerase class I |
| jty_rs13165 | JTY_2548 | Derived by automated computational analysis using gene prediction method: Protein Homology. |
| jty_rs11370 | qcrA | ubiquinol-cytochrome c reductase iron-sulfur subunit |
| jty_rs06340 | sigI | ECF RNA polymerase sigma factor SigI |
| jty_rs11505 | glnE | [glutamate--ammonia-ligase] adenylyltransferase |
| jty_rs03495 | atsD | arylsulfatase |
| jty_rs01410 | oplA | 5-oxoprolinase |
| jty_rs06075 | JTY_1174 | Derived by automated computational analysis using gene prediction method: Protein Homology. |
| jty_rs04295 | JTY_0834 | 4-amino-4-deoxychorismate lyase |
| jty_rs19900 | JTY_3877 | Cof-type HAD-IIB family hydrolase |
| jty_rs04425 |  | Derived by automated computational analysis using gene prediction method: Protein Homology. |
| jty_rs10100 | lppG | lipoprotein |
| jty_rs03290 | JTY_0643 | ribonuclease VapC5 |
| jty_rs20400 | JTY_3980 | single-stranded DNA-binding protein |
| jty_rs11105 | JTY_2154 | Derived by automated computational analysis using gene prediction method: Protein Homology. |
| jty_rs12080 | JTY_2342 | MarR family transcriptional regulator |
| jty_rs12705 | JTY_2463 | enoyl reductase |
| jty_rs14975 | xerC | tyrosine recombinase XerC |
| jty_rs00665 | JTY_0127 | Derived by automated computational analysis using gene prediction method: Protein Homology. |
| jty_rs10310 |  | DUF1490 domain-containing protein |
| jty_rs05665 | JTY_1092 | Derived by automated computational analysis using gene prediction method: Protein Homology. |
| jty_rs21275 |  | Derived by automated computational analysis using gene prediction method: Protein Homology. |
| jty_rs18675 | lppH | sensor domain-containing protein |
| jty_rs08365 | chaA | ionic transporter integral membrane protein ChaA |
| jty_rs06905 | hemK | peptide chain release factor N(5)-glutamine methyltransferase |
| jty_rs09285 | JTY_1802 | ferredoxin |
| jty_rs07400 | JTY_1429 | NAD(P)/FAD-dependent oxidoreductase |
| jty_rs18705 | ispD | 2-C-methyl-D-erythritol 4-phosphate cytidylyltransferase |
| jty_rs21570 |  | Derived by automated computational analysis using gene prediction method: Protein Homology. |
| jty_rs00555 | JTY_0106 | membrane protein |
| jty_rs06515 | tatB | twin-arginine translocase subunit TatB |
| jty_rs17705 | JTY_3447 | haloacid dehalogenase |
| jty_rs09510 | JTY_1849 | MerR family transcriptional regulator |
| jty_rs08845 | JTY_1715 | HNH endonuclease |
| jty_rs01530 | esxH | ESAT-6-like protein EsxH |
| jty_rs06050 | PPE16 | PPE family protein |
| jty_rs06050 | PPE16 | PPE family protein |
| jty_rs07340 | JTY_1418 | Derived by automated computational analysis using gene prediction method: Protein Homology. |
| jty_rs00285 |  | Derived by automated computational analysis using gene prediction method: Protein Homology. |
| jty_rs17580 | PPE56a | PPE family protein |
| jty_rs10740 | cobLa | precorrin-6Y methyltransferase |
| jty_rs16425 | hpx | alpha/beta hydrolase |
| jty_rs03540 | lpqP | Derived by automated computational analysis using gene prediction method: Protein Homology. |
| jty_rs05595 | JTY_1080 | NAD(P)-dependent oxidoreductase |
| jty_rs12145 | PE_PGRS41 | Derived by automated computational analysis using gene prediction method: Protein Homology. |
| jty_rs16985 | purE | 5-(carboxyamino)imidazole ribonucleotide mutase |
| jty_rs19180 | whiB4 | WhiB family transcriptional regulator |
| jty_rs18285 | yrbE4A | ABC transporter permease |
| jty_rs05765 | mca | mycothiol S-conjugate amidase |
| jty_rs20155 | whiB6 | transcriptional regulator |
| jty_rs13430 | apt | adenine phosphoribosyltransferase |
| jty_rs05115 | JTY_0990 | Derived by automated computational analysis using gene prediction method: Protein Homology. |
| jty_rs11295 | JTY_2191 | 1-acyl-sn-glycerol-3-phosphate acyltransferase |
| jty_rs10360 | JTY_2015 | SAM-dependent methyltransferase |
| jty_rs18105 | infA | translation initiation factor IF-1 |
| jty_rs11140 | ftsZ | cell division protein FtsZ |
| jty_rs09820 | JTY_1912 | polyisoprenoid-binding protein |
| jty_rs03805 | rpsE | 30S ribosomal protein S5 |
| jty_rs05170 | JTY_1001 | DUF1446 domain-containing protein |
| jty_rs16970 | JTY_3296 | CoA transferase |
| jty_rs11100 |  | antitoxin ParD2 |
| jty_rs07585 | PE16 | PE family protein |
| jty_rs11805 | JTY_2288 | Derived by automated computational analysis using gene prediction method: Protein Homology. |
| jty_rs13855 | hemE | uroporphyrinogen decarboxylase |
| jty_rs14435 | JTY_2804 | IS607 family transposase IS1602 |
| jty_rs07190 | JTY_1388 | Derived by automated computational analysis using gene prediction method: Protein Homology. |
| jty_rs10265 | JTY_1998 | chitinase |
| jty_rs02385 | JTY_0466 | toxin MazF1 |
| jty_rs10735 | cobLb | precorrin-6Y C5 15-methyltransferase (decarboxylating) subunit CbiT |
| jty_rs02070 | JTY_0404 | Derived by automated computational analysis using gene prediction method: Protein Homology. |
| jty_rs04175 | JTY_0810 | phosphoribosylformylglycinamidine synthase subunit PurS |
| jty_rs07540 | JTY_1457 | RNase adaptor protein RapZ |
| jty_rs06955 | atpD | ATP synthase subunit beta |
| jty_rs15620 | JTY_3032 | Derived by automated computational analysis using gene prediction method: Protein Homology. |
| jty_rs07380 | gmk | guanylate kinase |
| jty_rs03790 | rpsH | 30S ribosomal protein S8 |
| jty_rs14330 | JTY_2783 | Derived by automated computational analysis using gene prediction method: Protein Homology. |
| jty_rs07110 | JTY_1373 | rhomboid family intramembrane serine protease |
| jty_rs07345 | carA | carbamoyl-phosphate synthase small subunit |
| jty_rs10635 | fxsA | membrane protein FxsA |
| jty_rs16995 | JTY_3302 | GtrA family protein |
| jty_rs12490 | JTY_2419 | transglutaminase family protein |
| jty_rs09865 | JTY_1921 | Derived by automated computational analysis using gene prediction method: Protein Homology. |
| jty_rs17620 | JTY_3430 | Txe/YoeB family addiction module toxin |
| jty_rs19855 | JTY_3869 | membrane protein |
| jty_rs05725 | fadA3 | acetyl-CoA acetyltransferase |
| jty_rs06535 | lpqX | Derived by automated computational analysis using gene prediction method: Protein Homology. |
| jty_rs09895 | JTY_1927 | Derived by automated computational analysis using gene prediction method: Protein Homology. |
| jty_rs03780 | rplE | 50S ribosomal protein L5 |
| jty_rs12110 | JTY_2347 | Derived by automated computational analysis using gene prediction method: Protein Homology. |
| jty_rs06715 | pknH | serine/threonine protein kinase |
| jty_rs03820 | sppA | signal peptide peptidase SppA |
| jty_rs16525 |  | Derived by automated computational analysis using gene prediction method: Protein Homology. |
| jty_rs13835 | JTY_2681 | peptide-methionine (R)-S-oxide reductase |
| jty_rs11325 | JTY_2198 | GDP-mannose-dependent alpha-(1-6)-phosphatidylinositol monomannoside mannosyltransferase |
| jty_rs09540 | JTY_1855 | Derived by automated computational analysis using gene prediction method: Protein Homology. |
| jty_rs02095 | JTY_0408 | DUF3054 domain-containing protein |
| jty_rs00570 | rpmB1 | 50S ribosomal protein L28 |
| jty_rs13305 | JTY_2575 | Derived by automated computational analysis using gene prediction method: Protein Homology. |
| jty_rs13545 | JTY_2625 | pyridoxal 5'-phosphate synthase lyase subunit PdxS |
| jty_rs02815 | galE3 | UDP-glucose 4-epimerase |
| jty_rs16125 | moaB1 | 4a-hydroxytetrahydrobiopterin dehydratase |
| jty_rs04720 |  | Derived by automated computational analysis using gene prediction method: Protein Homology. |
| jty_rs13070 | JTY_2530 | Derived by automated computational analysis using gene prediction method: Protein Homology. |
