# Supplementary figures and images for: Small RNA Profiling in Mycobacterium Provides Insights Into Stress Adaptability
Source: Front Microbiol. 2021 Nov 4;12:752537. doi: 10.3389/fmicb.2021.752537 (PMC8600241; doi:10.3389/fmicb.2021.752537)

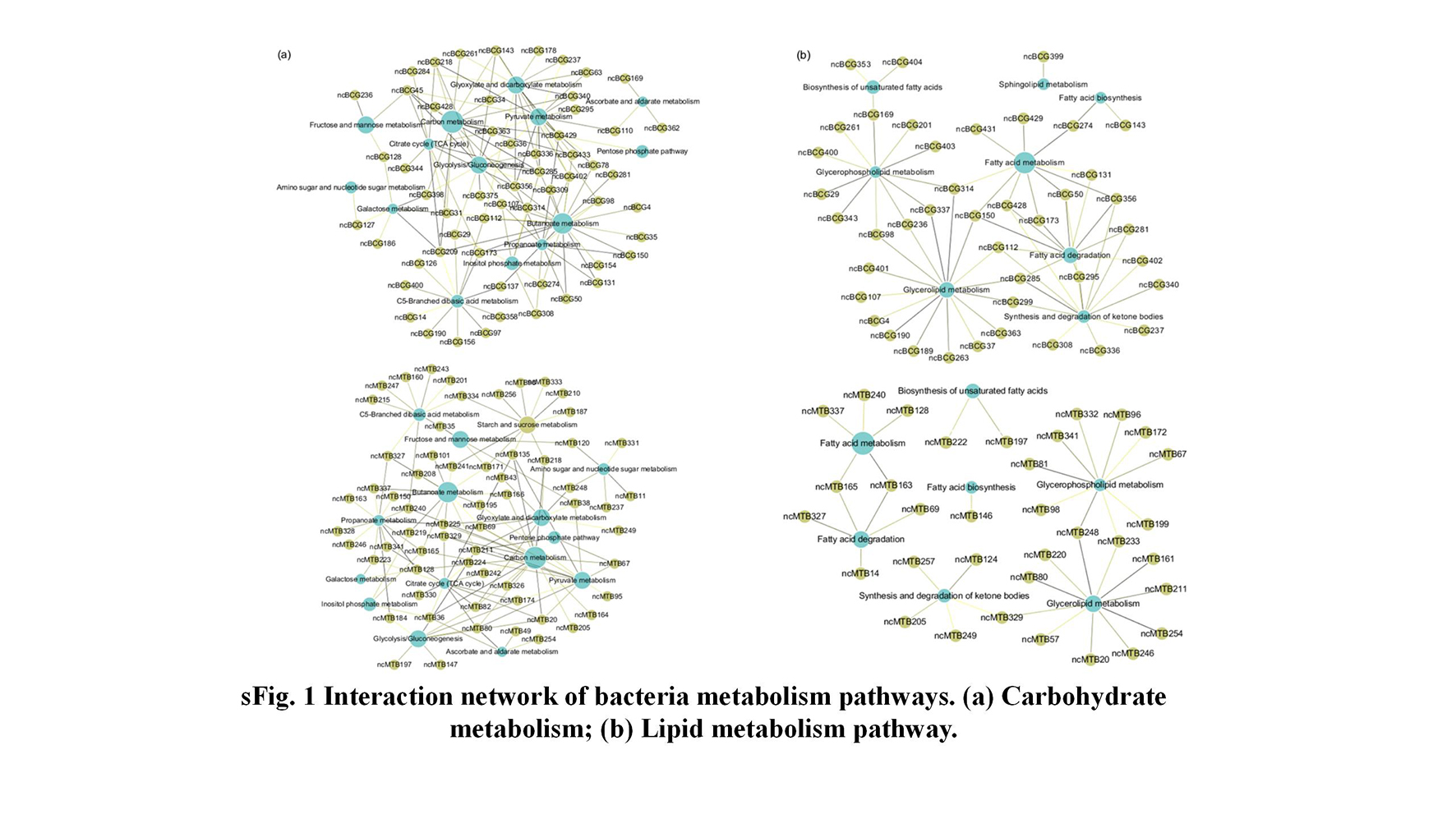

Supplement: Supplementary file 2 [file Image_1.JPEG]

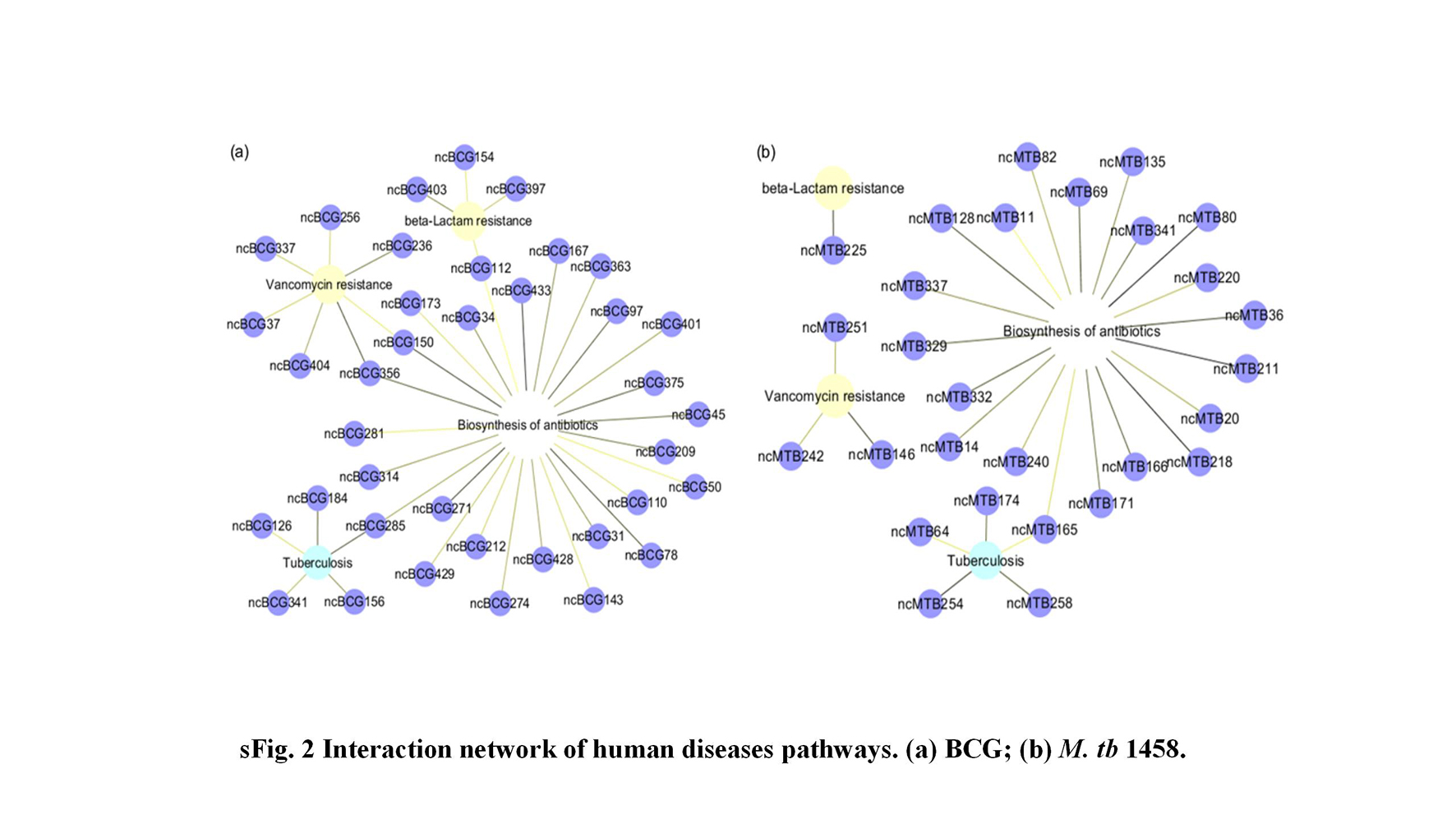

Supplement: Supplementary file 3 [file Image_2.JPEG]

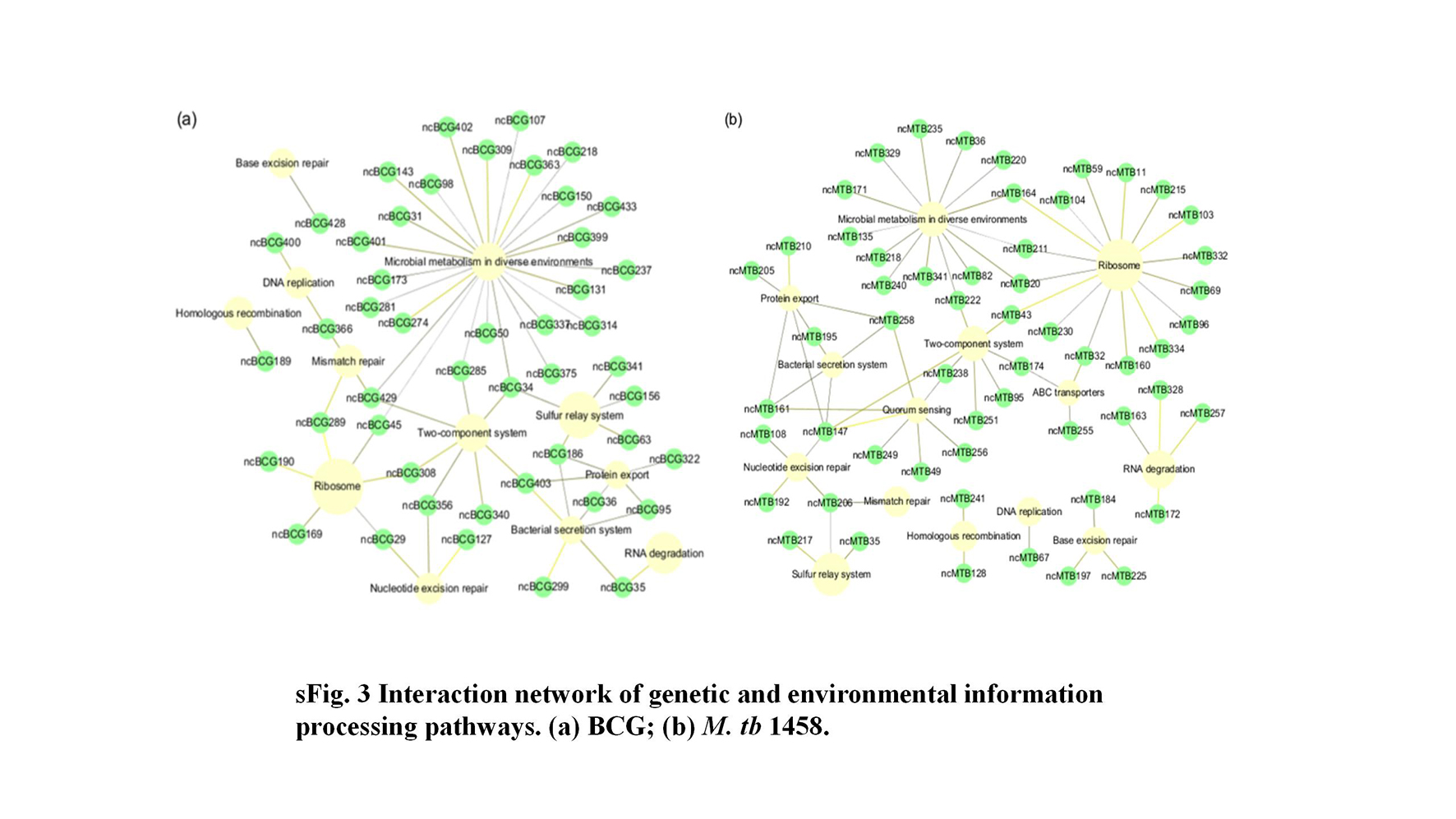

Supplement: Supplementary file 4 [file Image_3.JPEG]

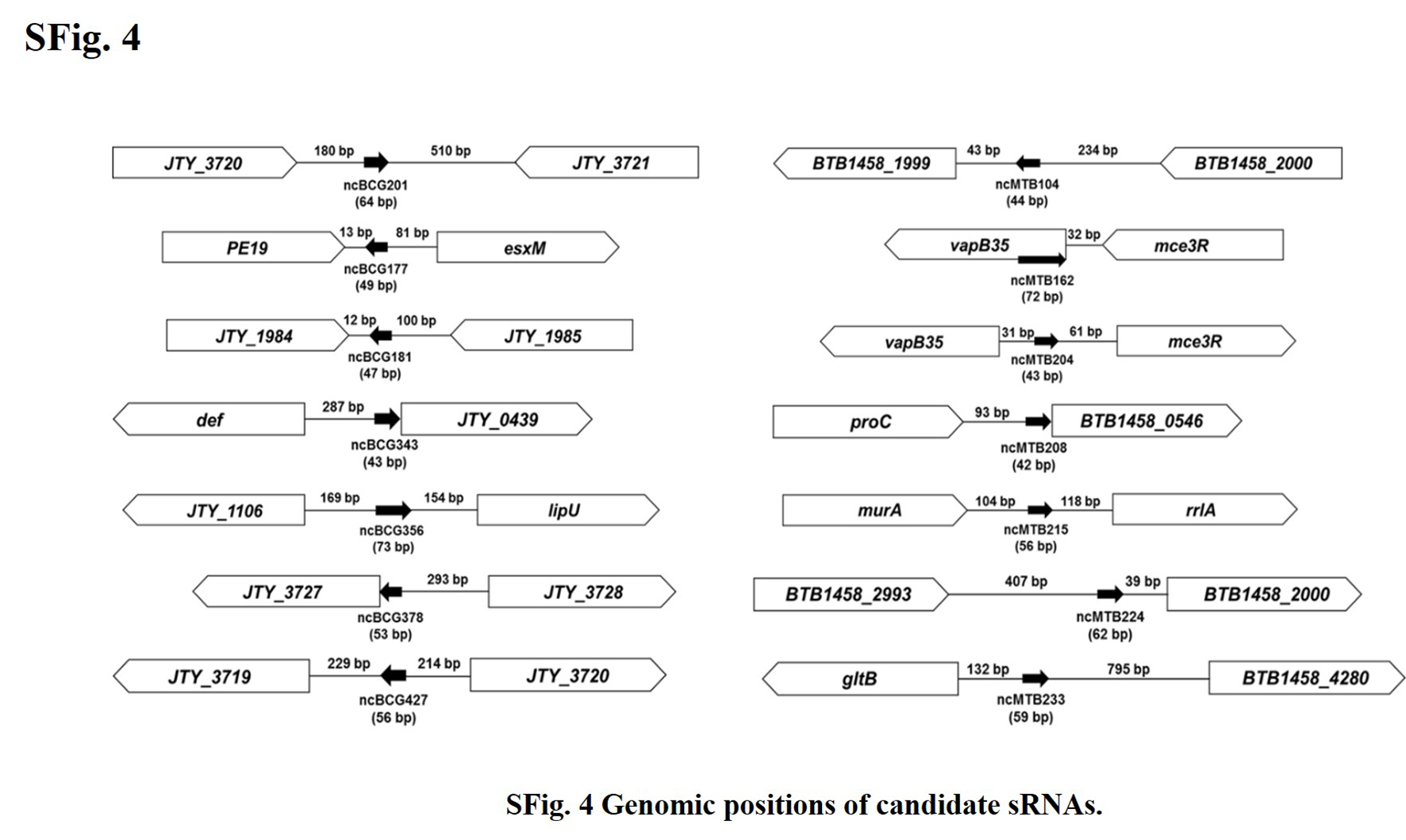

Supplement: Supplementary file 5 [file Image_4.JPEG]

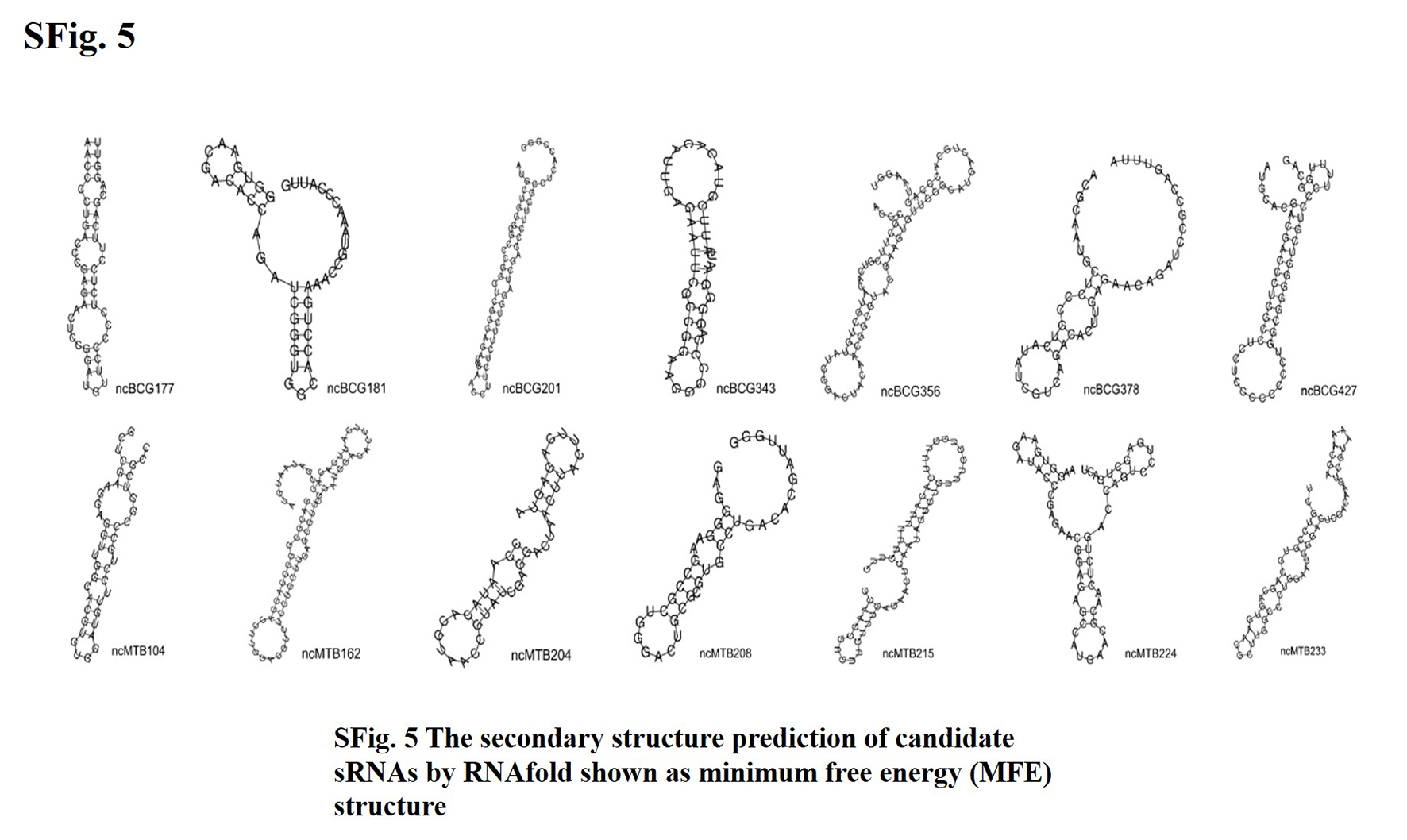

Supplement: Supplementary file 6 [file Image_5.JPEG]

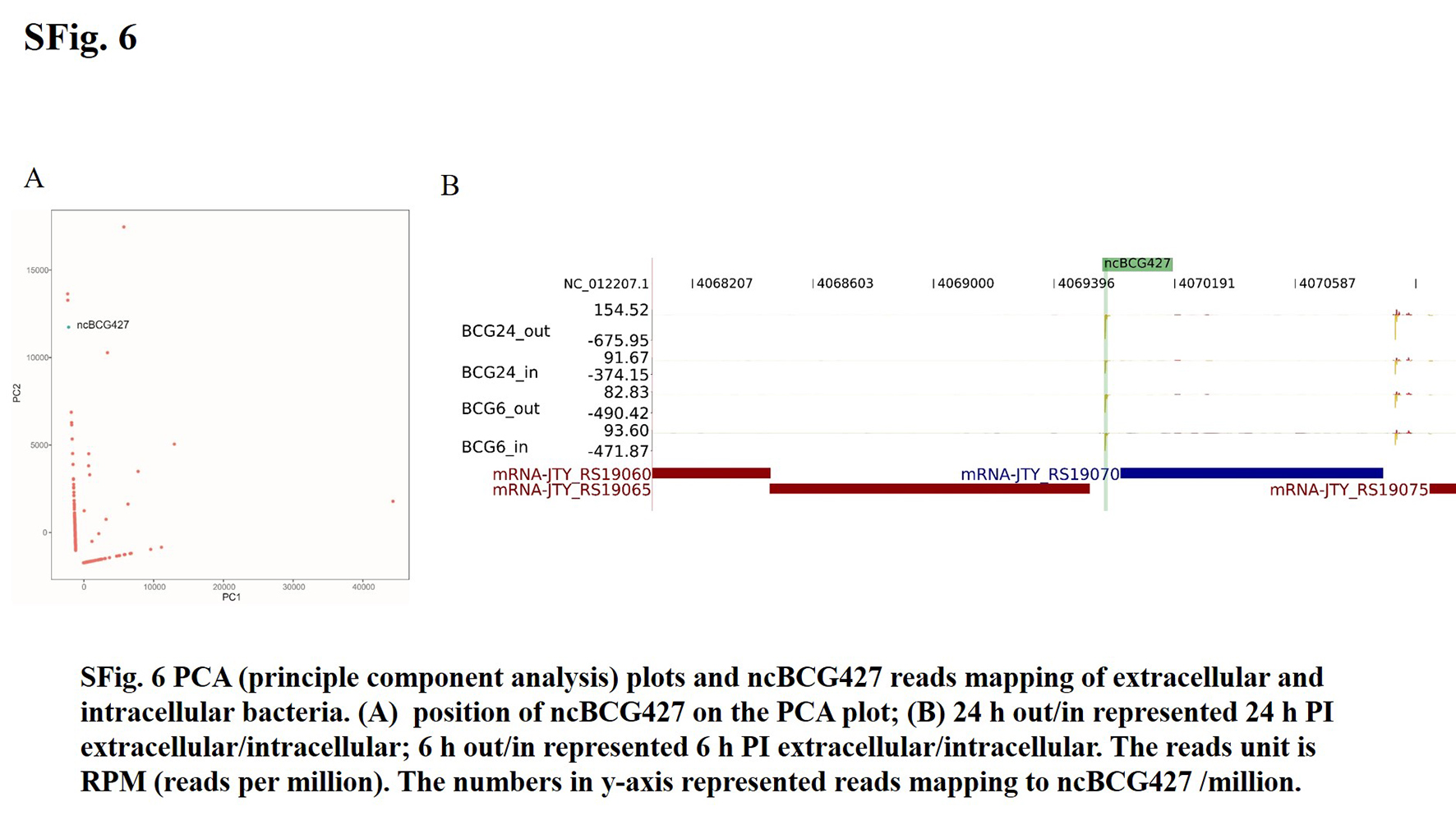

Supplement: Supplementary file 7 [file Image_6.JPEG]
